# Vitronectin Increases Vascular Permeability by Promoting VE-Cadherin Internalization at Cell Junctions

**DOI:** 10.1371/journal.pone.0037195

**Published:** 2012-05-11

**Authors:** Rong Li, Meiping Ren, Ni Chen, Mao Luo, Zhuo Zhang, Jianbo Wu

**Affiliations:** 1 Drug Discovery Research Center of Luzhou Medical College, Luzhou Medical College, Luzhou, Sichuan, People's Republic of China; 2 Dalton Cardiovascular Research Center, University of Missouri, Columbia, Missouri, United States of America; University of Bristol, United Kingdom

## Abstract

**Background:**

Cross-talk between integrins and cadherins regulates cell function. We tested the hypothesis that vitronectin (VN), a multi-functional adhesion molecule present in the extracellular matrix and plasma, regulates vascular permeability via effects on VE-cadherin, a critical regulator of endothelial cell (EC) adhesion.

**Methodology/Principal Findings:**

Addition of multimeric VN (mult VN) significantly increased VE-cadherin internalization in human umbilical vein EC (HUVEC) monolayers. This effect was blocked by the anti-α_V_β_3_ antibody, pharmacological inhibition and knockdown of Src kinase. In contrast to mult VN, monomeric VN did not trigger VE-cadherin internalization. In a modified Miles assay, VN deficiency impaired vascular endothelial growth factor-induced permeability. Furthermore, ischemia-induced enhancement of vascular permeability, expressed as the ratio of FITC-dextran leakage from the circulation into the ischemic and non-ischemic hindlimb muscle, was significantly greater in the WT mice than in the *Vn* ^−/−^ mice. Similarly, ischemia-mediated macrophage infiltration was significantly reduced in the *Vn* ^−/−^ mice vs. the WT controls. We evaluated changes in the multimerization of VN in ischemic tissue in a mouse hindlimb ischemia model. VN plays a previously unrecognized role in regulating endothelial permeability via conformational- and integrin-dependent effects on VE-cadherin trafficking.

**Conclusion/Significance:**

These results have important implications for the regulation of endothelial function and angiogenesis by VN under normal and pathological conditions.

## Introduction

Extracellular matrix (ECM) glycoproteins participate in many physiological and pathological processes, including ischemic vascular diseases, inflammation, tumor growth and metastasis. Endothelial cell (EC)-ECM interactions play a key role in cellular functions by binding multiple cell surface receptors and are critical for the activation of cell signal transduction pathways. One of the components of the ECM, vitronectin (VN), is a multifunctional glycoprotein that is in an inactive state in the plasma; however, the VN incorporated into the ECM is predominately multimeric, with exposed binding sites that interact with integrins and cell surface receptors that regulate cell adhesion and cellular motility. Levels of VN are increased in patients with various cardiovascular diseases, tumor growth and metastasis. Although mice with a genetic deletion of VN develop normal vasculature during embryogenesis, abnormal localization of VN is expected to lead to a conformational change that results in compromised vasculature, including pathological angiogenesis [1], [2], [3], [4]. The conformational change in VN followed by tissue injury or other pathological conditions are the key events in the regulation of cell adhesion [2], [3], [5].

Angiogenesis encompasses cell proliferation, migration, and differentiation. Loosening of cell-to-cell contacts is the key initial event that causes normally quiescent ECs to initiate angiogenesis. Cell-to-cell adhesion is mainly achieved by VE-cadherin, which is a critical EC-specific adhesion molecule. Activation of αvβ3 by integrin antagonists disrupt VE-cadherin and enhance vascular permeability [6], [7]. Recent studies have demonstrated that the binding of integrins to ECM components disrupts VE-cadherin regulated cell-to-cell adhesion by regulating the inactivation of Src [8], [9]. These studies suggest that cell-ECM interactions participate in the regulation of cell-to-cell contacts.

Previous studies have demonstrated that the αvβ3 integrin-VN interaction plays an important role in potentiating VEGF signaling by interacting with the integrin-VEGFR-2-Src signaling pathway [10], [11], [12]. VN has been shown to be involved in promoting transendothelial migration by interacting with leukocyte Mac-1 during adhesion and extravasation [13], [14]. Although ECM-derived VN multimerizes in response to various types of tissue injury, and this transition involves functional changes in the vessel, such as angiogenesis, the role of VN in VE-cadherin-mediated cell-to-cell contacts and vascular permeability remain unknown. In the present study, we evaluated whether VN is implicated in the regulation of cell-to-cell contacts and vascular permeability. We found that VN redistributes VE-cadherin by promoting internalization of VE-cadherin and increases vascular permeability. The process is inhibited by blocking the αvβ3 integrin and the Src kinase signaling pathway. Furthermore, in a mouse hindlimb ischemia model, we demonstrated that ischemia induced the multimerization of VN in ischemic tissues, and this multimerization was linked to ischemia-mediated vascular leakage.

**Figure 1:**
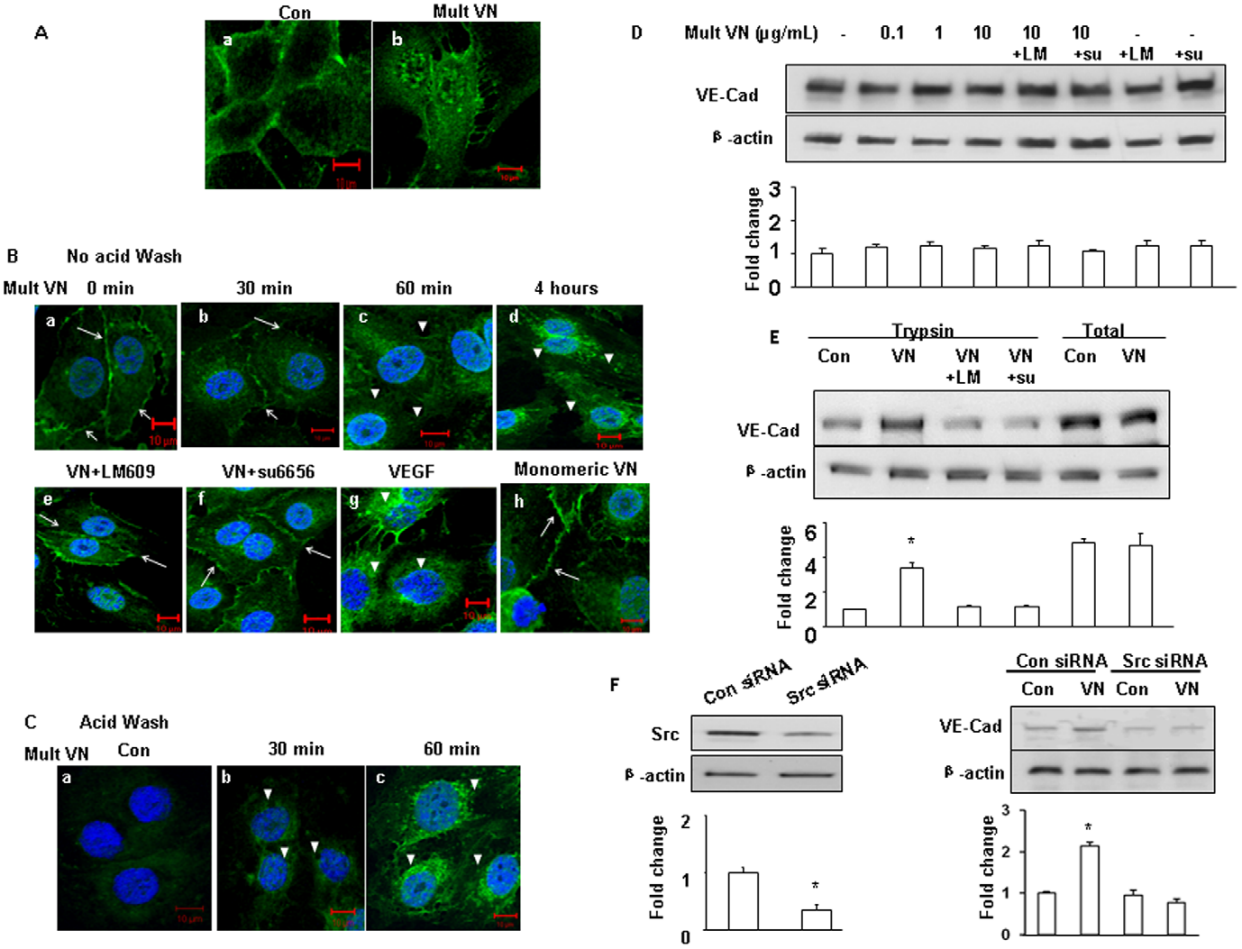
Mult VN promotes VE-cadherin internalization. (A) HUVECs were treated with mult VN (10 µg/mL). Confocal microscopy showed continuous and organized VE-cadherin staining, which is normally observed in ECs at cell-cell junctions (A-a). VN stimulation resulted in a disorganized pattern of VE-cadherin distribution at the membrane and led to intracellular accumulation of VE-cadherin (A-b); (B). To further determine whether cell surface-derived VE-cadherin was internalized into intracellular compartments, cells were prepared without acid washing and analyzed using confocal microscopy as described in *Methods*. A time-dependent internalization of VE-cadherin was observed (upper panels, B-a-d). Pretreatment with LM 609 (LM) and su6656 (su) prevented VN-mediated VE-cadherin internalization (B-e, f, lower panels). VE-cadherin internalization was not observed after treatment with mono VN (10 µg/mL)(B-h). VEGF (50 ng/mL) as a positive control (B-g). (C). Cells were prepared by acid washing and analyzed using confocal microscopy and cell surface VE-antibody was removed with a low pH acid wash. VN treatment increased the intracellular accumulation of VE-cadherin internalized from the cell surface (C-b, c) relative to no treatment (C-a). Nuclei are stained with DAPI. The scale bars represent 10 µm. Arrows: cell surface; Arrowhead: intracellular accumulation. (D). HUVEC monolayers were treated with VN at the indicated dose. Cells were lysed using RIPA buffer for western blotting analysis of total VE-cadherin. (E). Cells were treated with trypsin/EDTA, pelleted and lysed with RIPA for western blotting analysis of intracellular VE-cadherin. (F). HUVECs were transfected with Src siRNA or scrambled siRNA (con siRNA). The efficiency of Src protein expression knock-down was analyzed using immunoblot (left panel). Cells were treated with trypsin/EDTA, pelleted and lysed with RIPA for western blotting analysis of intracellular VE-cadherin (right panel). β-actin was used as a loading control.

**Figure 2:**
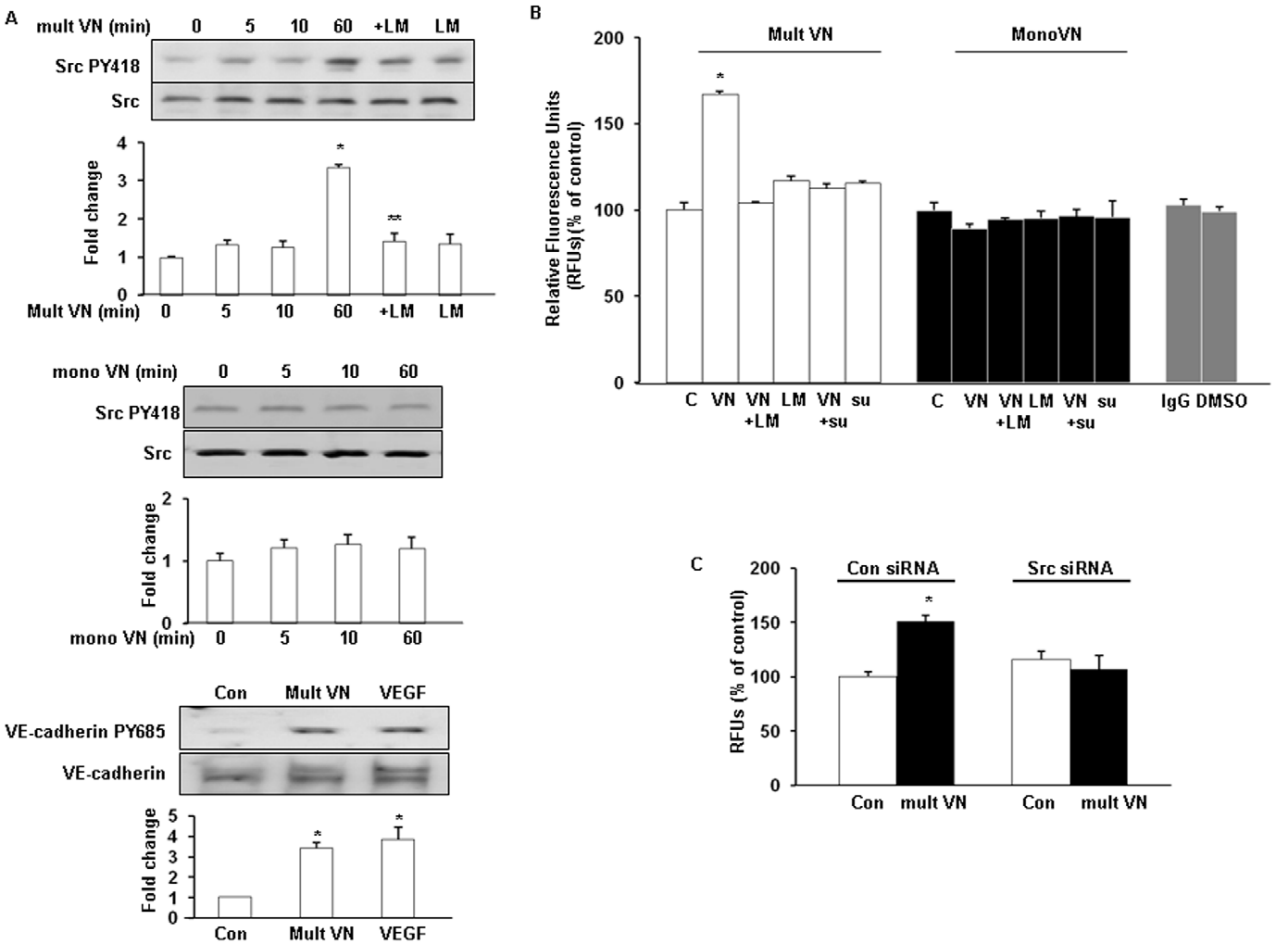
Mult VN activates Src and increases vascular permeability. (A). HUVECs were pretreated with LM609 (LM) (25 µg/mL) for 30 min before mult or mono VN (10 µg/mL) treatment. Phosphorylation of Src (PY418) was determined by western blotting analysis(upper and middle panel); Mult VN stimulates tyrosine phosphorylation of VE-cadherin in HUVECs. Cells were incubated with mult VN (10 µg/mL) for 60 min or VEGF(50 ng/mL) for 30 min. Cell extracts were subjected to immunoprecipitation with antibody against VE-cadherin. Precipitated protein was analyzed by Western blot using anti-phosphotyrosine antibody. The same blot was then subsequently reprobed with antibody to VE-cadherin. (B). Mult VN, not mono VN increases vascular permeability in vitro using the permeability assay. HUVEC monolayers were pretreated with LM609 (LM) (25 µg/mL) and su6656 (su) (1 µM) for 30 min before mult or mono VN (10 µg/mL) treatment. Permeability is represented by relative fluorescence units measured by the flux of FITC-Dextran across the monolayer of HUVECs. Immunoglobulin G (IgG) and dimethyl sulfoxide (DMSO) vehicle were used as the controls. (C). Knockdown of Src with siRNA blocked VN-induced vascular permeability. The results are expressed as the mean ± SEM. * p<0.05 *vs*. control.

**Figure 3:**
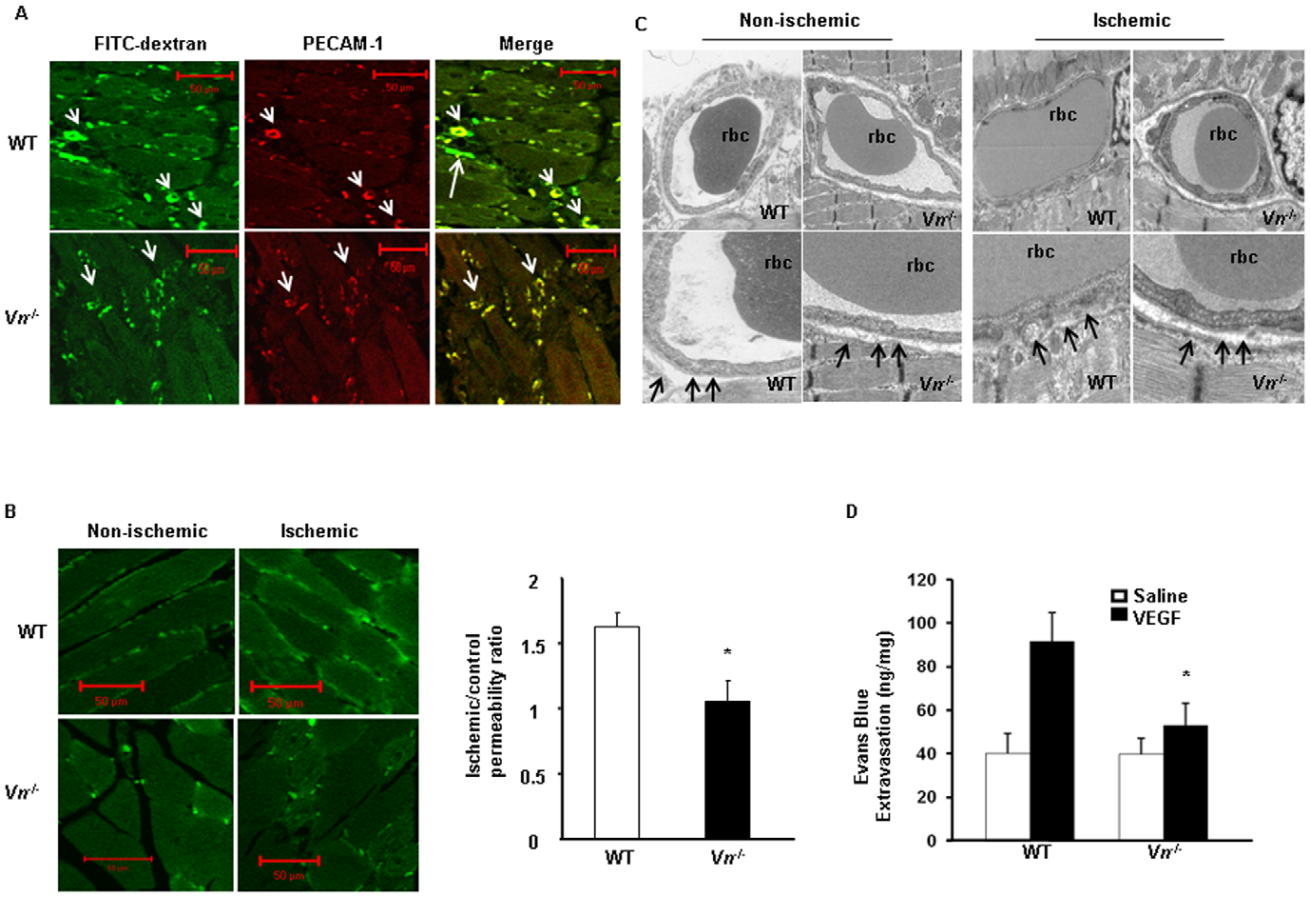
*Vn^−/−^* mice impaired vascular permeability. (A). Whole-mount FITC-dextran angiograms in the WT and the *Vn^−/−^* mice at day 7 in a mouse hindlimb ischemia model. Dextran leakage was more marked in the wild-type mice (upper panels, arrows) than in the *Vn^−/−^* mice in ischemic gastrocnemius muscles. Capillaries were visualized by red Cy3-conjugated PECAM-1 antibodies. (B). Fluorescent intensities of cross-sections of non-ischemic (R2) and ischemic muscles (R1) were measured, and the R1/R2 ratio was used to assess the level of extravasation of FITC-dextran. Permeability was reduced in the gastrocnemius muscles in the *Vn^−/−^* mice compared to the WT mice (n = 6, *p*<0.05). (C). Electron microscopy of capillaries in the ischemic gastrocnemius muscles showed a diffuse and irregular basement membrane without distinct boundaries in WT mice in contrast to those of the *Vn^−/−^* mice (upper panel, scale in 2 µm; lower panel is higher magnification. (D). The miles assay was performed with 50 ng/mL of VEGF and saline in the right and left ears, respectively, in the WT and *Vn^−/−^* mice. Evan's blue dye extravasation was quantified with a spectrophotometer. The results are expressed as the mean ± SEM. * p<0.05. (n = 6 for each strain).

**Figure 4:**
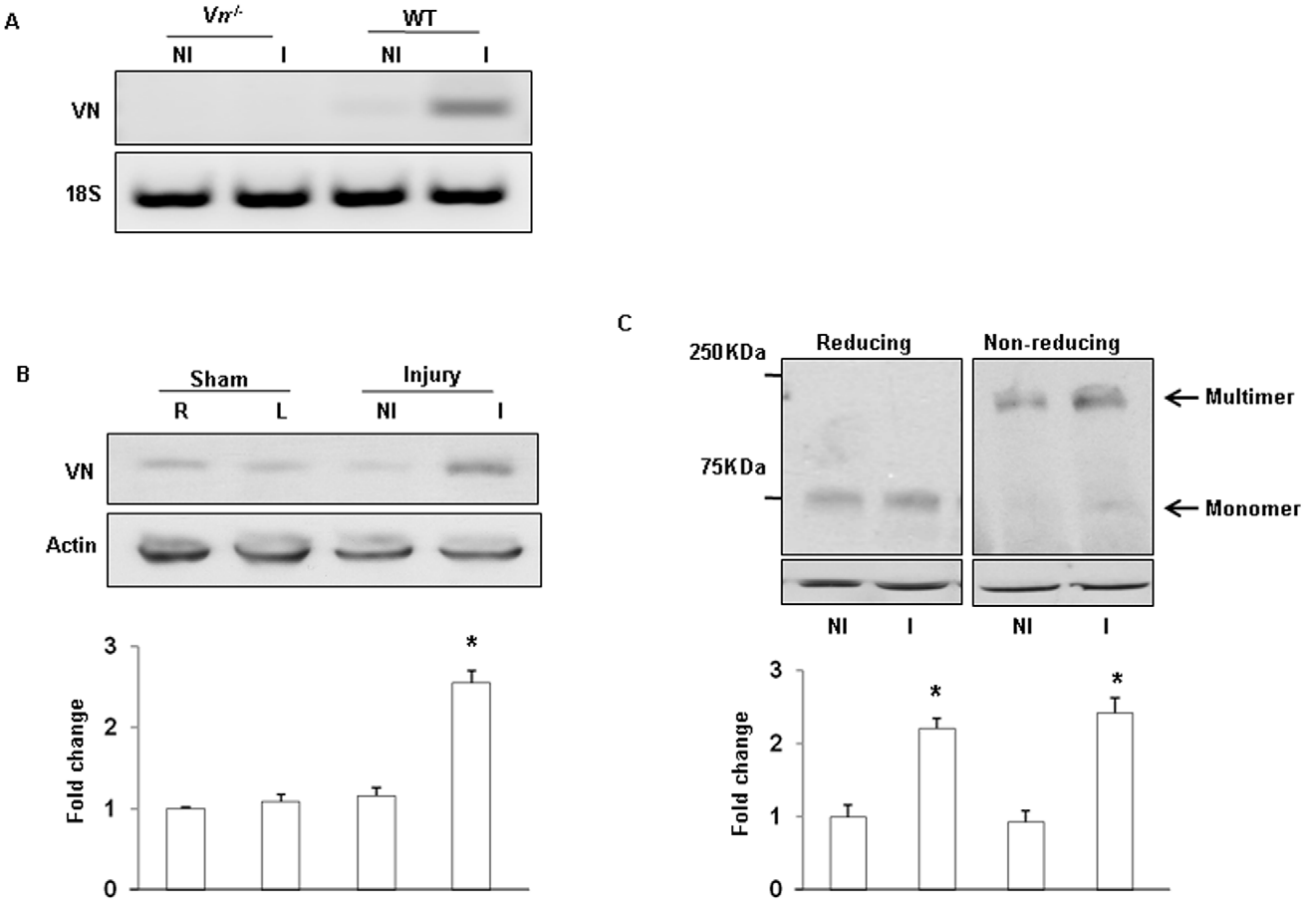
Increased multimerization of VN in the tissue after ischemia. (A) and (B). RT-PCR and western blot analysis of VN from the gastrocnemius muscles indicated that ischemia induced VN expression in the WT mice (n = 6). R: Right leg; L: Left leg, NI: Nonischemic; I: Ischemic. (C). An equal amount of gastrocnemius muscle lysates (each 20 µg) were subjected to SDS-PAGE under nonreducing and reducing conditions and were analyzed by immunoblotting. The mobility of molecular weight standards for SDS-PAGE is indicated. Data are presented as fold changes (n = 9; *p<0.05 vs. control).

**Figure 5:**
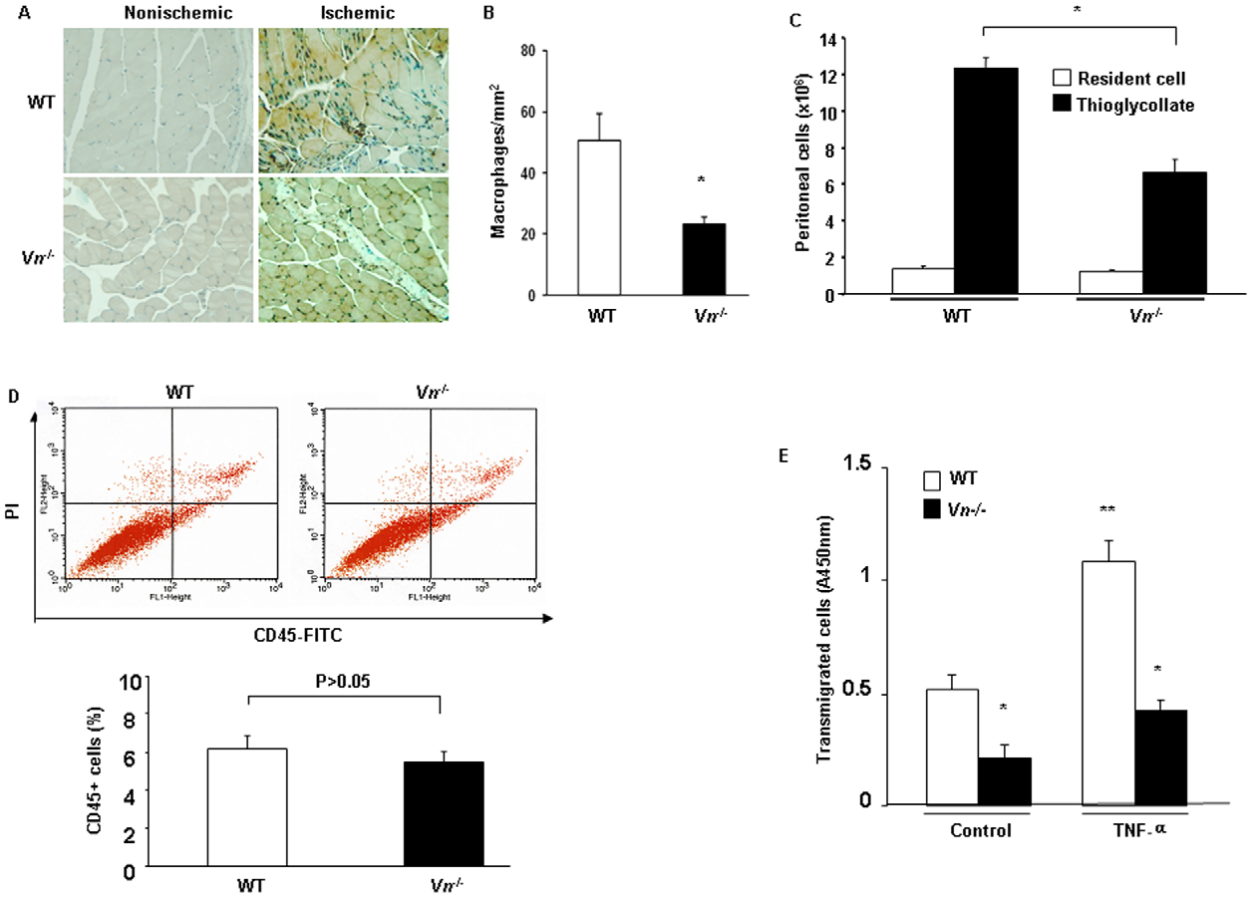
*Vn^−/−^* mice reduced inflammatory cells infiltration. (A) Recruitment of macrophages in response to ischemia as determined by the Mac-3 antibody. Representative images of macrophages in nonischemic and ischemic muscles in the WT and *Vn^−/−^* mice on day 7 after femoral artery ligation are shown. (B) The number of Mac3-positive cells was quantified. Data are the mean ± SEM from 10 fields per section (3 sections/mouse and n = 6 for each strain). *p<0.05. (C) The number of thioglycollate-elicited mouse peritoneal macrophages was quantified. The results are expressed as the means ± SEM. * p<0.05. (n = 6 for each strain). (D) The number of CD45-FITC- and PI- positive cells in peripheral blood was analyzed by flow cytometry after hindlimb ischemia by staining with CD45-FITC and PI (Propidium iodide). The results are expressed as the means ± SEM. * p>0.05. (n = 6 for each strain). (E). Transendthelial migration of murine mononuclear cells with TNFα of ECs. Murine aortic ECs were grown to confluency on cell culture inserts. ECs were then treated with 20 ng/mL TNFα for 4 hrs. Murine mononuclear cells were added on the endothelial layer for 4 hrs. Migrated cells were stained and measured by OD 450 nm according to the assay instructions. The results are expressed as the mean ± SEM. *p<0.05 vs. WT; **p<0.05 vs. control. Data shown are representative of three independent experiments.

## Results

### VN induces VE-cadherin internalization

VE-cadherin is a major junctional molecule that mediates cell–to-cell adhesion [15], [16]. There is evidence that binding of the integrins-ECM components perturbs VE-cadherin-mediated adherens junctions [8], [9]. To examine the role of VN in VE-cadherin-mediated cell-to-cell contacts, we investigated the impact of VN on VE-cadherin distribution. In confluent monolayers of untreated HUVECs, cell surfaces displayed intact VE-cadherin staining (Figure 1A-a). In contrast, continuous VE-cadherin staining in cell-to-cell contacts was disrupted by mult VN after 4 hrs of treatment (Figure 1A-b), whereas fibronectin and type I collagen did not markedly alter the levels of intracellular VE-cadherin (data not shown). Furthermore, in a time-response curve, mult VN promoted the rapid intracellular accumulation of VE-cadherin (Figure 1B-a-d). The internalization of VE-cadherin induced by mult VN could be prevented by pretreatment with LM609, an αvβ3 integrin antibody, and by su6656, a Src family kinase inhibitor (Figure 1B-e, f). Here, VEGF was used as a positive control (Figure 1B-g), as it has been observed to promote VE-cadherin internalization [15], [17]. Importantly, mono VN (10 µg/mL) had no effect on VE-cadherin (Figure 1B-h), which indicated that mult VN is critical for VE-cadherin internalization at cell-to-cell contacts.

To directly observe intracellular VE-cadherin staining, a specific immunofluorescence protocol for fixation and sample preparation was used in the visualization [15], [18]. The cells were acid washed to remove the cell surface VE-cadherin bound antibodies. Increased accumulation of intracellular VE-cadherin was observed with VN treatment compared to the control (Figure 1C-b-c). Internalization of the VE-cadherin caused by mult VN was also demonstrated using western blot analysis. Mult VN did not have an effect on the VE-cadherin expression in total cell lysates from HUVEC monolayers at the indicated concentrations (Figure 1D). To further distinguish the accumulation of the cell surface and intracellular VE-cadherin, HUVECs were trypsinized to remove cell surface cadherin. Increased VE-cadherin internalization in the intracellular compartment was observed with trypsin detachment (Figure 1E), and the internalization was inhibited with pretreatment with LM609 and su6656. Furthermore, the role of Src in VE-cadherin internalization was confirmed using Src siRNA (Figure 1F and Supplemental Figure S2). These results indicated that VN promotes VE-cadherin internalization and may be involved in the activation of the αvβ3 integrin-Src signaling pathway.

### Mult VN, but not mono VN activates Src and increases vascular permeability

Activation of Src induces ECM-mediated disruption of VE-cadherin at cell-cell adherents [8], [9]. Therefore, we investigated if such a complex may be involved in internalization of VE-cadherin and the increase of vascular permeability with VN treatment. We compared the ability of mult VN and with mono VN to activate Src kinase by examining the phosphorylation status of Src. As shown in Figure 2A (upper and middle panel), phosphorylation of Src in HUVECs was observed with mult VN but not with mono VN, and its activation was inhibited by LM609, which suggests that the mult VN-induced activation of Src correlates with the binding of αvβ3 integrin. These findings suggested that mult VN may regulate VE-cadherin internalization through binding of the αvβ3 integrin to Src, leading to Src activation. We also examined the effect of mult VN on the phosphorylation of VE-cadherin, as shown in Figure 2A (lower panel), mult VN stimulated the tyrosine phosphorylation of VE-cadherin, indicating that the phosphorylation of VE-cadherin by mult VN maybe involved in the internalization of VE-cadherin. We next performed an *in vitro* permeability assay. As shown in Figure 2B, mult VN-induced permeability of the EC monolayer was inhibited by pretreatment with LM609 and su6655. The role of Src in VN-mediated vascular permeability was further confirmed with the knockdown of Src by siRNA (Figure 2C).

### VN deficiency reduced vascular permeability

To gain insight into the role of VN in ischemia-mediated permeability *in vivo*, we examined vascular integrity in a mouse hindlimb ischemia model. Whole-mount staining revealed that one day after FITC-dextran injection of mice, there was an higher level of perivascular leakage and diffuse deposition of FITC in the ischemic gastrocnemius muscles of the WT mice compared with that of the *Vn^−/−^* mice. PECAM-1 immunostaining was performed to visualize capillary formation (Figure 3A). The fluorescent intensities of cross-sections that were assessed by confocal microscopy were significantly greater in the WT mice than in the *Vn^−/−^* mice (1.64×10^5^±0.09 *vs.* 1.05×10^5^±0.15/um^2^ ischemic/non-ischemic; n = 6; p = 0.02; Figure 3B). Ultrastructural examination of the ischemic gastrocnemius muscles revealed that a diffuse and irregular EC lining without distinct boundaries was found in the WT mice but not in the *Vn^−/−^* mice (Figure 3C). These changes were associated with EC swelling and increased endothelial permeability, as described previously [19], [20]. Furthermore, we used the Miles assay to measure vascular leakage in the skin of *Vn^−/−^* and WT mice. As shown in Figure 3D, VEGF-mediated hyperpermeability was significantly impaired in the *Vn^−/−^* mice compared with that in the WT mice. Taken together, these results indicated that VN increases vascular permeability *in vivo*.

### Increased multimerization of VN after ischemia

To determine whether the increased permeability is associated with VN deposition and conformational change after ischemia, we examined VN expression in response to ischemic injury in a mouse hindlimb model. As shown in Figure 4A, VN gene expression in ischemic gastrocnemius muscles was determined by RT-PCR. Ischemia significantly increased the expression of VN in ischemic gastrocnemius muscles at day 7 compared with non-ischemic gastrocnemius muscles in WT mice (n = 9/group). Western blot analysis demonstrated that VN protein was markedly increased in ischemic gastrocnemius muscles at day 7 after ischemic injury (Figure 4B). Because mult VN is the active form of this protein and is the form that interacts with the ECM, we next examined the conformational changes of VN in ischemic tissues. As shown in Figure 4C, under reducing conditions, VN in ischemic gastrocnemius muscles was detected as a ∼75 kDa protein, and no high molecular weight forms were detected. Ischemia-induced VN was further analyzed under nonreducing conditions, and the intensities of the multimer bands in GM VN were remarkably increased with ischemia, which is characteristic of mult VN [21], [22]. *Vn^−/−^* mice showed no detectable VN (data not shown). These observations suggest that multimerization of VN was induced by ischemia.

To determine the role of VN in ischemia-induced angiogenesis, we measured capillary density by staining ischemic tissue with anti-PECAM-1 antibody. Supplemental Figure S1 shows that PECAM-1-positive capillary density was significantly reduced in the ischemic tissue of *Vn^−/−^* mice compared with WT mice.

### VN deficiency suppress inflammatory cells infiltration

To determine whether VN-induced vascular permeability could be related to inflammatory cell infiltration in ischemia, we examined the infiltration of macrophages in ischemic muscles. Infiltration of macrophages into the ischemic hind limb was determined by immunostaining with the anti-Mac3 antibody. No macrophages were detected in nonischemic muscles (Figure 5A). Ischemia induced an increase in infiltration of macrophages in the WT mice relative to the *Vn^−/−^* mice at day 7 post femoral ligation (50.70±8.58 *vs.* 23.31±2.32 macrophages/mm^2^, n = 6, p<0.05)(Figure 5B). Next, we examined whether leukocyte infiltration was reduced in another model of inflammatory response in *Vn^−/−^* mice. The number of leukocytes recruited into the peritoneal cavity was counted at 72 hours after injection of thioglycollate, a stimulatory agent to induce non-infectious peritoneal inflammation for elicitation of macrophages. The number of resident cells in the peritoneal cavity was unchanged between WT and *Vn^−/−^* mice, however, thioglycollate-elicited cells were significantly reduced in *Vn^−/−^* mice compared with that in WT mice (Figure 5C) (6.60±0.77 *Vs.* 12.33±0.56, n = 5, p<0.01). FACS analysis showed that the number of CD45-FITC- and PI-positive cells in peripheral blood was unchanged between WT and *Vn^−/−^* mice after hindlimb ischemia (Figure 5D). Further, we assessed in vitro transendthelial migration (TEM) of murine mononuclear cells. The results showed that *Vn^−/−^* mononuclear cells had significantly impaired TEM in both untreated and TNFα-treated groups as compared to WT mononuclear cells (Figure 5E). Thus, VN deficiency was associated with reduced transendothelial migration of leukocytes.

## Discussion

The binding of VN to EC surfaces is mediated by the u-PA receptor and integrins [23], [24]. It is well established that VE-cadherin is critical for the stability of cell-to-cell junctions. At least three molecular pathways are involved in regulating this process: i) degradation of VE-cadherin at cell-to-cell contacts, ii) tyrosine phosphorylation of VE-cadherin [25], and iii) internalization of VE-cadherin at cell-to-cell contacts [15], [18]. Here, we show that mult VN increases vascular permeability by promoting internalization of VE-cadherin at cell-to-cell contacts in a Src-dependent mechanism.

The role of integrin-ECM interaction in controlling vascular permeability has been studied in different ways *in vitro* and *in vivo* [6], [26], [27]. In normal physiological conditions, the application of RGD peptides or αvβ3 antagonists had an inhibitory effect on vascular permeability, which indicated that binding of the αvβ3 integrin-VN maintains normal endothelial barrier function. Importantly, tissue injury or activation of inflammatory cells usually results in the release of enzymes capable of exposing cryptic RGD fragments from ECM components, including multimerization of VN and fibronectin [3], [28]. We observed that VN stimulation resulted in the rapid internalization of VE-cadherin at cell-to-cell junctions. These findings indicated that VN can disrupt cell-to-cell contacts by promoting the internalization of VE-cadherin and can cause the loss of VE-cadherin from the cell surface. Our report supports the observations that matricryptic peptide fragments of VN have vascular permeability effects, as previously discussed in the vascular responses to tissue injury [3], [29].

We found that VN deficiency impaired vascular permeability in the ischemic hindlimb, which suggests that VN is necessary for ischemia-induced hyperpermeability after arterial occlusion. However, the role of VN in normal physiological regulation is somewhat controversial. Previously, Wu et al. found that integrin binding to VN maintains the barrier function in isolated porcine coronary venules [26], furthermore, treatment of the venules with VN did not significantly alter basal permeability, which provides information that is relevant to the physiological mechanisms controlling microvascular exchange. We speculate that physiological regulation of VN is largely related to an inactive mono form. In contrast, tissue injury or other pathological alterations could cause VN conformational change, which may promote vascular leakage, as observed in our hindlimb ischemia model.

We demonstrated that VN induced the activation of Src through αvβ3 integrin. Both pharmacological inhibition and Src siRNA significantly inhibit VN-induced internalization of VE-cadherin and endothelial vascular permeability. EC adhesion to provisional ECM components, such as fibronectin and collagen I, has been shown to disrupt VE-cadherin at the cell-to-cell contacts in a Src dependent mechanism [8], [9]. Previous work has demonstrated that the binding of αvβ3 integrin to VN potentiates the activation of Src kinase, which is linked to EC migration [10], [11], [12]. This mechanism correlates with the binding of Src to the cytoplasmic domain of both the α and β subunits [29]. In the present study, we confirmed that VN promotes Src phosphorylation in ECs, and the αvβ3 integrin is required for the activation of Src. In addition to binding to integrins on the cell surface, VN also interacts with uPAR. This binding is important in controlling extracellular proteolysis and cellular adhesion. The mechanism by which Src mediates VE-cadherin internalization remains unclear; however, it is interesting to note that the internalization of VE-cadherin is largely dependent on binding of αvβ3 integrin by VN, which in turn, triggers the crosstalk between the αvβ3 integrin and Src.

We have investigated the synthesis and conformational changes of VN after femoral ligation in the plasma and tissue of ischemic mice. In this study, we showed that VN was highly upregulated in ischemic muscles. Most importantly, conformational changes in VN were induced by ischemia. The increased local changes in the multimerization of VN, either produced locally or derived from vascular leakage of circulating VN, is likely attributable to an increase in vascular permeability during ischemia.

The transendothelial migration of leukocytes has been shown to be enhanced by vasoactive substances that disrupt VE-cadherin at endothelial junctions and increase vascular permeability [30], [31], [32]. In fact, recent studies have shown that VN contributes to neutrophil chemotaxis [13], [14]. In the present study, the number of infiltrated leukocytes was lower in the *Vn^−/−^* mice than in the WT mice. These results suggest that VN-mediated VE-cadherin localization seems to be important for leukocyte migration across the endothelium.

Our findings suggest that the binding of to αvβ3 integrin mult VN promotes the internalization of VE-cadherin via the activation of Src activation. The observed increased in permeability induced by mult VN is consistent with internalization of VE-cadherin and its disappearance from cell-to-cell contacts. Multimerization of VN plays an important role in ischemia-induced angiogenesis *in vivo*. The mechanism is likely related to inflammatory cell infiltration by VN-mediated VE-cadherin at cell-to-cell contacts and vascular permeability.

## Materials and Methods

### Reagents

Human multimeric VN (Mult VN) and human monomeric VN (Mono VN) were obtained from Molecular Innovations (Southfield, MI). Human multimeric VN (Mult VN) was prepared from fresh human plasma using urea as a denaturant. Human recombinant vascular endothelial growth factor (VEGF) was purchased from R&D Systems. The inhibitor of the Src family kinase su6656 was obtained from Calbiochem (La Jolla, CA). LM609 (anti-αvβ3 integrin antibody) was purchased from Chemicon. The following antibodies were used: rabbit anti-VE-cadherin (Sigma), rabbit anti-c-Src, and rabbit anti-phosphor-Tyr-418-Src (Biosource, QCB, Camarillo, CA).

### Cell Culture

Human umbilical vein endothelial cells (HUVECs, Cascade Biologics) were grown in Medium 200 (Cascade Biologics) supplemented with LSGS. Mouse EC were isolated from mouse aortas and grown in culture as described previously [33]. Mouse mononuclear cells were isolated from peripheral blood according to the manufacturer's protocol by using Histopaque (Sigma) centrifugation. Cells were resuspended in DMEM plus 0.25% BSA for transmigration experiments.

### Immunofluorescence and VE-cadherin Internalization assay

Cells were grown on coverslips and were treated with VN. Cells were fixed in cold methanol followed by extraction in 0.5% Triton X-100. Distribution of VE-cadherin was detected using rabbit anti-human VE-cadherin (Santa Cruz Biotechnology, Inc.). The secondary antibodies used were goat ant-rabbit IgG AlexaFluor 546-conjugated antibody (Molecular Probes, Invitrogen). Images were analyzed with a Zeiss LSM 510 2-photon confocal system.

For immunofluorescence studies of the internalization of VE-cadherin, cells were treated using a protocol adapted from previous publications [15], [18]. Briefly, cells were incubated with rabbit anti-human VE-cadherin for 1 hour. Cells were then treated with VN. To remove the cell-surface bound antibodies while retaining the internalized antibodies, cells were acid washed for 30 min in phosphate-buffered saline (PBS), pH 2.7, containing 25 mM glycine and 3% bovine serum albumin. The cells were rinsed, fixed, and processed for immunofluorescence as described above. All pictures are representative of 3 independent experiments.

### Miles vascular permeability assay

The miles assay was performed as previously described [34]. Briefly, mice were administered Evans blue dye, and VEGF (300 ng in 15 µl) or saline was then injected subcutaneously into the dorsal surface of the right and left ears, respectively. After 30 minutes, mice were euthanized and their ears removed, oven-dried at 55°C, and weighed. The Evans blue dye was then extracted from the ears using 500 µl of formamide for 24 hours at 55°C, and the absorbance of extracted dye was measured at 630 nm.

### Mouse ischemic hindlimb model

Age-matched C57BL/6J mice were purchased from Jackson Labs. C57BL/6J-congenic VN-deficient (*Vn* ^−/−^) mice were a gift from Dr. David Ginsburg, University of Michigan [35]. All animal care and experimental procedures complied with the *Guide for the Care and Use of Laboratory Animals* published by the US National Institutes of Health (NIH publication no. 85-23, revised 1996), and experiments were approved by the Office of Animal Resources of the University of Missouri. Mice were anaesthetized with sodium pentobarbital (60 mg/kg body weight, intraperitoneally) or isoflurane (5%, by inhalation) and a single subcutaneous dose of buprenorphine hydrochloride (0.1 mg/kg) for analgesia. Additional sodium pentobarbital (12 mg/kg body weight) or 5% isoflurane was given as needed to maintain anaesthesia. The depth of anaesthesia was evaluated by pinching the pedal withdrawal reflex. Mice were euthanized by cervical dislocation at the end of the experiment while still anaesthetized. Mice were subjected to unilateral hindlimb ischemia as previously described [36]. Mouse hindlimb protein samples were immunoblotted according to the method of Laemmli under reducing or nonreducing conditions [37], [38] and the RT-PCR analysis [39] for mouse VN. In some experiments, mouse hindlimbs were used for immunostaining.

### Fluorescent dextran injection

To assess the ischemia-mediated permeability of microvessels, mice were subjected to femoral artery ligation for 7 days. A 250 µl of 0.25 mg/mL solution of FITC-conjugated dextran (75 kDa, Sigma) was injected into the tail vein 1 day before sarcrifice. All gastrocnemius muscles were fixed for whole mounting and immunostaining using cy3-conjugated mouse PECAM-1 antibody (Sigma). The amount of FITC-dextran in the gastrocnemius muscles was determined with confocal microscopy. The ratio of the FITC intensities in the ischemic to the intensities in the non-ischemic muscle was calculated for each mouse.

### siRNA

The Src siRNA target sequence was CTCCATGTGCGTCCATATTTA. Cells were transfected with 0.5 µg of siRNA duplexes using the RNAi Starter Kit (Qiagen). Transfected cells were incubated in culture medium for 48 hr and were harvested for detection of Src by western blot. A second set of cells was used for the *in vitro* permeability assay.

### RT-PCR

Total RNA was extracted from the cells using TRIzol reagent (Invitrogen) and digested with DNase I (Ambion). Reverse transcriptase (RT)-polymerase chain reaction (PCR) was performed using SuperScript 1-step RT-PCR (Invitrogen). Mouse VN and 18 S mRNA levels were determined by reverse transcription–polymerase chain reaction (RT-PCR). The band intensity of the RT-PCR products was quantified using the Fluochem 8800 Imaging system (Aloha Innotech).

### Immunoblotting

HUVECs were harvested in RIPA buffer (Sigma), and the total protein was subjected to 4–20% SDS-polyacrylamide gel. Transferred membrane was probed with the rabbit anti-VE-cadherin antibodies (Santa Cruz Biotechnology) as described. In some experiments, HUVECs were rinsed and were incubated in trypsin-EDTA at 37°C for 5 min to remove cell surface VE-cadherin as previously described [15], [32]. Trypsin was inactivated using normal growth medium and the cells were recovered by centrifugation. Cell pellets were lysed for immunoblotting. For control, parallel cultures were harvested in RIPA buffer without trypsinization.

### *In vitro* permeability assay

Endothelial permeability was analyzed *in vitro* by diffusing of 75 kDa fluorescein isothiocyanate (FITC)-dextran (Sigma) through the endothelial monolayer [18]. HUVECs were grown to confluence on transwell inserts (Costar, Cambridge, MA, USA). The cells were pretreated with the Src inhibitor, su6655 (1 µM), and the αvβ3 integrin antibody, LM609 (25 µg/mL) for 30 minutes. VN (10 µg/mL) was added to medium for 4 hours. Medium containing 75 kDa FITC-dextran was then loaded in the upper compartment of the transwell. The amount of FITC-dextran that diffused through the endothelial monolayer into the lower compartment was measured using a microplate reader (Molecular Devices, Sunnyvale, CA).

### *In vitro* leukocytes transmigration assay

Migration assays were performed with QCM™ leukocyte transendothelial migration assay kit (Millipore, USA) using the supplier's protocol. Briey, murine aortic EC were grown to conuence on Matrigel-coated Transwell filters and treated with TNF-α overnight. The lower chamber was filled with assay medium (DMEM plus 0.25% BSA) plus 5 ng/ml IL-8 as chemoattractant. EC were washed twice with assay medium and subsequently incubated with 1×10^5^ mononuclear cells at 37°C, 10% CO_2_ for 4 h. 20 µl of WST-1 solution was added to each well. The plate was incubated for three hours at 37°C and 5% CO_2_. Conversion of WST-1 solution was measured by absorbance at 450 nm.

### FACS Analysis

To quantify inflammatory cells in peripheral blood, WT and *Vn^−/−^* mice (n = 4 each) were subjected to hindlimb ischemia. Peripheral blood was suspended in PBS containing 1% BSA and was subjected to flow cytometry. Inflammatory cells were analyzed using anti-mouse CD45 antibody and propidium iodide. Cells were finally fixed with 1% paraformaldehyde (Ph 7.5) in PBS and analyzed by flow cytometry.

### Transmission electron microscopy

Each harvested gastrocnemius muscle was fixed with 3% glutaraldehyde. After postfixation with 2% osmium tetroxide, the tissues were dehydrated in a graded series of ethanol and were embedded in Epon 812. The ultra-thin sections were cut, stained with uranyl acetate, and examined under a JEOL 1400 EX transmission electron microscope (Nippon Denshi Ltd., Tokyo, Japan) at 80 kV.

### Immunostaining

Gastrocnemius muscle sections (10 µm) were mounted on glass slides. Immunostaining was performed as described previously [18]. Slides were blocked with BSA and were incubated with rabbit IgG raised against mouse PECAM-1 (Santa Cruz Biotechnology). Negative controls were incubated without the primary antibody. The secondary antibody was biotinylated goat IgG raised against rabbit IgG (Santa Cruz Biotechnology). Washed slides were incubated with Vectastain Elite ABC reagent (Vector Laboratories), developed using DAB substrate (Vector Laboratories), and counterstained with hematoxylin. Images were captured with a 40× objective lens using an Olympus (DP70) microscope. Vessel density was calculated as vessel number per field and plotted as mean ± SEM.

### Thioglycollate-induced peritonitis

Macrophages were obtained by flushing the peritoneal cavity with PBS 4 days after intraperitoneal injection with 1 mL of a 3% thioglycollate solution (Difco Laboratories, Sparks, MD). Collected elicited-macrophages from peritoneum were counted by microscopy in WT and *Vn* ^−/−^ mice.

#### Statistical Analysis

Data are expressed as the mean ± SEM from experiments that were performed at least in triplicate. Groups were compared using Student's 2-tailed *t* test.

## Supporting Information

Figure S1 **Impaired capillary formation in** ***Vn^−/−^*** **mice.** (A). Anti-PECAM-1 staining was performed to examine capillary density in ischemic and nonischemic gastrocnemius muscles. Representative capillary density was enhanced in ischemic tissue in WT mice, but not *Vn^−/−^* mice. (B). Capillary density was expressed as the number of PECAM-1-positive cells per high power filed (×400). Data are presented as fold changes. n = 6 for each stain. *p<0.05 vs. *Vn^−/−^*. Scale bar denotes 5 µm.
(TIF)

Figure S2 **Knock down of Src blocks mult VN-induced VE-cadherin internalization.** HUVECs were transfected with Src siRNA or con siRNA. Cells were treated with mult VN (10 µg/mL) for 4 hrs. Cells were prepared for anti-VE-cadherin staining and analyzed using confocal microscopy as described in *Methods*. Nuclei are stained with DAPI. The scale bars represent 10 µm. Arrows: cell surface; Arrowhead: intracellular accumulation. Representative confocal fluorescence microscopy images of 3 experiments are shown.
(TIF)
