# Supplementary figures and images for: Vitronectin Increases Vascular Permeability by Promoting VE-Cadherin Internalization at Cell Junctions
Source: PLoS One. 2012 May 11;7(5):e37195. doi: 10.1371/journal.pone.0037195 (PMC3350505; doi:10.1371/journal.pone.0037195)

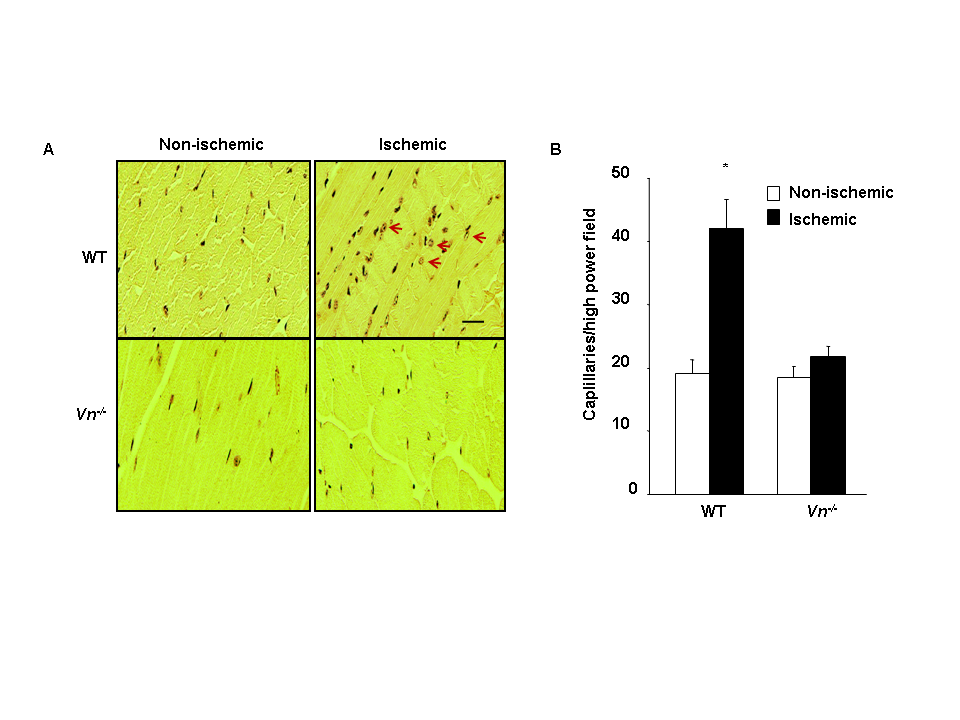

Supplement: Figure S1 — Impaired capillary formation in Vn−/− mice. (A). Anti-PECAM-1 staining was performed to examine capillary density in ischemic and nonischemic gastrocnemius muscles. Representative capillary density was enhanced in ischemic tissue in WT mice, but not Vn−/− mice. (B). Capillary density was expressed as the number of PECAM-1-positive cells per high power filed (×400). Data are presented as fold changes. n = 6 for each stain. *p<0.05 vs. Vn−/−. Scale bar denotes 5 µm. (TIF) [file pone.0037195.s001.tif]

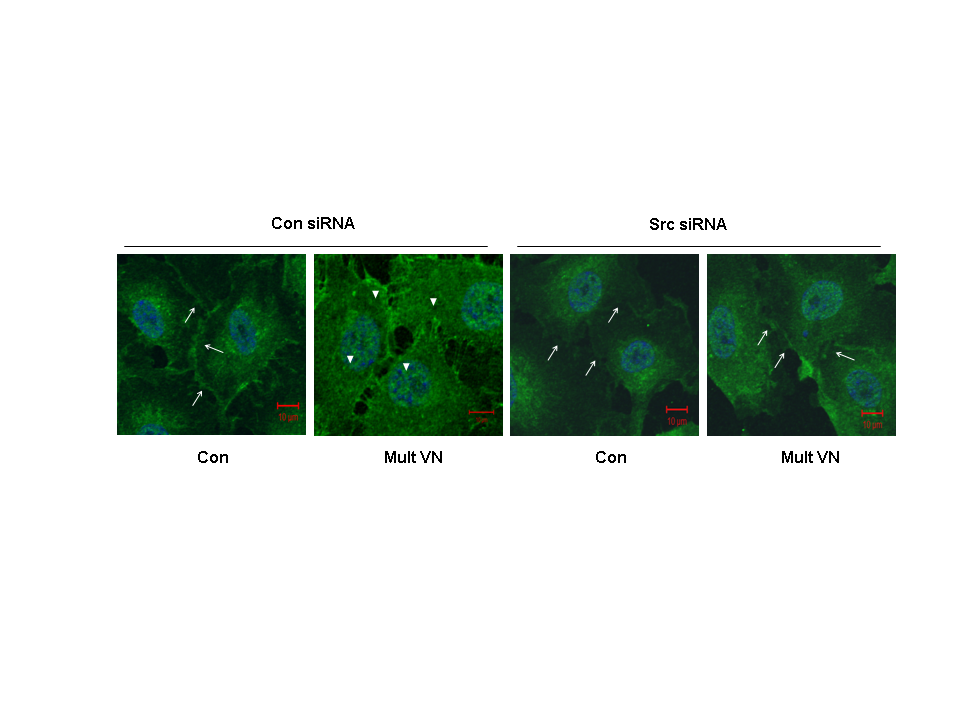

Supplement: Figure S2 — Knock down of Src blocks mult VN-induced VE-cadherin internalization. HUVECs were transfected with Src siRNA or con siRNA. Cells were treated with mult VN (10 µg/mL) for 4 hrs. Cells were prepared for anti-VE-cadherin staining and analyzed using confocal microscopy as described in Methods . Nuclei are stained with DAPI. The scale bars represent 10 µm. Arrows: cell surface; Arrowhead: intracellular accumulation. Representative confocal fluorescence microscopy images of 3 experiments are shown. (TIF) [file pone.0037195.s002.tif]
